# Chain Gangs: New Aspects of Hyaluronan Metabolism

**DOI:** 10.1155/2012/893947

**Published:** 2011-12-18

**Authors:** Michael Erickson, Robert Stern

**Affiliations:** Department of Basic Biomedical Sciences, Touro College of Osteopathic Medicine, 230 West-125th Street, New York, NY 10027, USA

## Abstract

Hyaluronan is a matrix polymer prominent in tissues undergoing rapid growth, development, and repair, in embryology and during malignant progression. It reaches 10^7^ Daltons in size but also exists in fragmented forms with size-specific actions. It has intracellular forms whose functions are less well known. Hyaluronan occurs in all vertebrate tissues with 50% present in skin. Hyaluronan provides a scaffold on which sulfated proteoglycans and matrix proteins are organized. These supramolecular structures are able to entrap water and ions to provide tissues with hydration and turgor. Hyaluronan is recognized by membrane receptors that trigger intracellular signaling pathways regulating proliferation, migration, and differentiation. Cell responses are often dependent on polymer size. Catabolic turnover occurs by hyaluronidases and by free radicals, though proportions between these have not been determined. New aspects of hyaluronan biology have recently become realized: involvement in autophagy, in the pathology of diabetes., the ability to modulate immune responses through effects on T regulatory cells and, in its fragmented forms, by being able to engage several toll-like receptors. It is also apparent that hyaluronan synthases and hyaluronidases are regulated at many more levels than previously realized, and that the several hyaluronidases have functions in addition to their enzymatic activities.

## 1. Introduction

Hyaluronan (HA, hyaluronic acid) is a glycosaminoglycan (GAG) polymer that is a major component of the extracellular matrix (ECM) [1, 2]. It can occur as a high-molecular-weight (HMW) polymer, reaching >10^7^ Da, but also exists in much smaller forms. The myriad of shorter HA chains occurs with activities that appear to be size specific. HA is major component of the ECM, particularly prominent during embryogenesis, in tissues undergoing rapid growth and development, during repair and regeneration, and in association with aggressive malignancies. Recent evidence indicates that HA also exists in an intracellular form. However, its functions therein are not as well established. Here, we attempt to summarize recent findings of this widely distributed GAG and to correlate specific functions with size and location of this intriguing carbohydrate chain.

## 2. Hyaluronan, a Highly Ironic Acid

### 2.1. Overview

HA is a strictly alternating disaccharide of N-acetylglucosamine and glucuronic acid connected by *β*1-4 and *β*1-3 glycosidic bonds, respectively. It is the only GAG that is not sulfated and without a covalent attachment to a proteoglycan core protein. It participates in many disparate processes, such as cell motility, tissue proliferation, embryonic development, malignant progression and metastasis, wound healing, and angiogenesis. HA is extruded into the extracellular space through the plasma membrane as it is being synthesized. Otherwise, the cell would become engorged with this huge space-occupying polymer.

 HA has a relatively small volume of solvent water, but because of its great negative charge at neutral pH, it has an immense volume of aqueous domain that accompanies the molecule. This forces open tissue spaces through which cells can travel. The HA molecule, by binding to cell surface receptors that interact with the cytoskeleton and that stimulate signal transduction pathways, endow cells with motility [3].

### 2.2. Hyaluronan of the Extracellular Matrix

Hyaluronan, in addition to occurring in a great number of sizes, also exists in a number of different forms in the vertebrate ECM. It can be firmly intercalated within a proteoglycan complex such as in cartilage, where it is held by electrostatic interactions by aggrecan and link protein with an avidity that approaches that of the avidin: biotin complex. HA can loosely be associated with an array of proteoglycans in loose connective tissues and in tissues undergoing rapid proliferation, development repair, and regeneration. It has a key role in organization of the entire ECM. HA can occur bound to the surface of cells by a number of membrane receptors.

 The argument can be made that there are actually two forms of ECM, a widespread general intercellular matrix, and a more delicate pericellular matrix, often referred to as a glycocalyx. HA is a major component of both of these matrices, but is the greater portion of the total matrix of the glycocalyx. The glycocalyx of endothelial cells extends into the vascular lumen. The HA of the endothelial glycocalyx controls permeability and the diffusion of small solutes [4–6]. The functional lumen of the vascular system is far smaller than reflected in formalin-fixed tissue sections, occupying less that 25% of the apparent volume. There are proteoglycan- and HA-rich fronds that extend into the lumen, referred to by physiologists as “Duling's Lawn.” This “lawn” disappears in a murine model, albeit transiently, following an *in vivo *injection of hyaluronidase (B.R. Duling, personal communication).

 One of the several nonenzymatic functions of the cell-surface-bound hyaluronidase, Hyal2, is formation of the glycocalyx [7]. In structures such as bone, tendon, cartilage, and loose connective tissues, the ECM constitutes a major proportion of total tissue mass. In the intimate pericellular matrix that envelops individual cells, HA remains consistently the major ECM component and is critically involved in mechanotransduction, in rounding of cells during mitosis, in adhesion and deadhesion of cells, and in cell locomotion [8].

 How HA becomes distributed into the two ECM components following synthesis is probably managed by the binding proteins that interact and decorate the polymer, including cell-bound receptors, and proteins with a wide range of avidity for HA. Such binding occurs in response to specific tissue needs and stresses, particularly in inflammation.

 The polymer also exists in a freely circuiting form in lymphatics and in the cardiovascular system where it can be decorated by a number of binding proteins termed the hyaladherens, attached by both electrostatic and covalent interactions. These include cross-linking proteins such as tenascin, TSG-6 (tumor-necrosis-factor-stimulated gene 6), inter-alpha-trypsin inhibitor, pentraxin, and thrombospondin. Even in these circulating forms, there is probably some structural organization conferred by these associated proteins [9, 10].

 A universal transitional ECM has recently become identified that enwraps muscle bundles, consisting of HA, tenascin, and fibronectin. This complex, synthesized by the deep fascia, supports limb regeneration in the salamander [11]. In higher vertebrates, this same complex may support muscle regeneration and repair. In surgery, it is customary in procedures involving limbs, muscles, and nerves, to provide a “fascial flap.” This improves recovery, but no mechanism has ever been formulated as to how this occurs. The fascial “transitional matrix,” rich in HA, may provide such a mechanism.

### 2.3. Hyaluronan Cables

Hyaluronan can also exist in cable forms, as has recently been documented [12], reflecting the remarkable diversity of this simple molecule. Incorporation of the heavy chain of I*α*I (inter-alpha-trypsin inhibitor) into bundles of HA facilitates formation of these cables, becoming simultaneously adhesion depots for inflammatory cells. These HA cables may be able to modulate the intensity of the inflammatory response. In marked contrast, the HA of the pericellular glycocalyx is not able to bind inflammatory cells, and actively excludes them. Of interest is that I*α*I is not associated with the HA of the glycocalyx and may therefore be the key mechanism. Other HA-binding proteins, such as versican, are found in both cables and in the glycocalyx.

### 2.4. Intracellular Hyaluronan

Intracellular HA has been well documented since 1999 [13–15], but its precise functions remain elusive. Many obstacles hinder precise localization, the most important being masking of HA by HA-binding macromolecules termed hyaladherens. These include proteoglycans, glycoproteins, and HA receptors. Despite that problem, many studies indicate that HA is indeed present intracellularly. Such HA is more prominent in proliferating cells where it is associated with microtubules, RHAMM (receptor for HA-mediated motility), and the mitotic spindle, suggesting functions associated with some aspects of mitosis and motility [14]. Intracellular HA is also more prominent in association with the stem cell niche, and with cellular stress responses such as inflammation. However, precise molecular mechanisms of such HA actions remain elusive.

 Other conundrums associated with the distinction between intracellular and extracellular HA are the mode of synthesis, and distribution. Is all HA that is synthesized, secreted by cells through the plasma membrane and intracellular HA is then taken up by cells secondarily? Or are there two pathways of HA synthesis, one for export and another for maintenance within the intracellular compartment. Intracellular HA is found intracellularly in the cytoplasm, nucleus, and nucleolus. What are the mechanisms for the distribution to HA to each specific location?

## 3. The Hyaluronan Synthases

Three isoforms of a single enzyme synthesize HA, dual-headed transferases that utilize as substrates alternately UDP-glucuronic acid, and UDP-N-acetylglucosamine. These are membrane proteins, located within and on the inner surface of the plasma membrane. The HA synthases are themselves synthesized in the endoplasmic reticulum in an inactive form, transported through the Golgi in an inactive form, and are then inserted into the plasma membrane. The enzymes must be activated, and they then begin to add alternatively the glucuronic acid and N-acetylglucosamine units to the reducing end of the growing polymer from their respective UDP precursors.

 The active sites of the HA synthase enzymes protrude from the inner surface of the cell membrane. The newly made HA is extruded through the plasma membrane into the extracellular space, either into the ECM or on to the cell surface as it is being synthesized, permitting unconstrained polymer growth without destruction of the cell.

 The three synthase genes in the mammalian genome code for HAS1, 2, and 3. These homologous isoenzymes each contain seven membrane-associated regions, and a central cytoplasmic domain possessing consensus sequences that are substrates for phosphorylation by protein kinase C. Even though they have 50–71% amino acid sequence identity, they are located on three separate chromosomes, human chromosomes 19, 8, and 16, respectively. This suggests gene duplications with divergent evolution. They are differentially regulated, each producing a different size polymer [16, 17], with independent gene regulation and kinetics.

 A number of biologicals are directly able to regulate expression of HA synthase mRNA, including upregulation by bFGF [18, 19], and several proinflammatory cytokines [20] as well as suppression, by glucocorticoids [21, 22]. The latter may account for the atrophic changes observed following long-term glucocorticoid administration. Enhanced expression occurs also through lactoferrin, which also upregulates transcription of a type-1 collagen gene, COL1A1 [23]. These together suggest a mechanism by which wound healing is stimulated.

 The activity of the HAS proteins can also be modulated by posttranslational modifications, such as ubiquitination and dimerization [24]. Aberrant splice variants of HAS1 are able to modulate expression of the normally spliced variant [25]. Abnormalities of the HA synthase enzymes have been associated with transformation and tumor metastases [2, 26]. Inherited and acquired variations in the HAS1 gene can contribute to disease progression in multiple myeloma and in Waldenstrom's macroglobulinemia [27]. Stimulation of the HA synthases in cultured cells in the presence of increased glucose promotes the deposition of an HA-rich matrix [28, 29]. This matrix is highly adhesive for monocytes and promises to provide new insights into diabetes. The HAS genes and their gene products are obviously tightly controlled at a vast number of levels, reflecting the extreme importance of HA in so many biological systems and the involvement in so many disease states [30].

## 4. Hyaluronan Catabolism

The breakdown of HA in tissues can occur by two entirely separate mechanisms, either enzymatically, by a class of enzymes known as hyaluronidases, or by cleavages that are nonenzymatic oxidation reactions.

### 4.1. The Hyaluronidases

Hyaluronan metabolism is extraordinarily active in vertebrates. The average 70 Kg individual has approximately 15 g of HA, 5 g of which turns over daily. HA has a half-life of 3–5 minutes in the circulation and just days in most tissues [31, 32]. Why the body eliminates HA so rapidly is not known. Hyaluronan is a scavenger of free radicals and reactive oxygen species. Removing these hazardous molecules and other toxic materials quickly may explain the rapid rate of HA elimination. The function of HA as a mechanism for the removal of toxins has not been explored but deserves attention.

 There are two separate mechanisms for removing HA from the body, local cleavage reactions, catalyzed by free radicals and enzymatic activity, and by whole body elimination through the action of liver and kidneys. Ligation of the hepatic or renal arteries causes an instant rise in circulating HA levels [33], demonstrating the efficiency of such mechanisms. Clinically, this explains also the high levels of HA in patients in renal or hepatic failure, as well as in patients following liver or kidney transplants experiencing transplant rejection.

 There are several classes of enzymes referred to as hyaluronidases: (1) prokaryotic enzymes that are eliminases, yielding disaccharides as the predominant products, (2) the eukaryotic enzymes that use a hydrolytic cleave reaction, adding water across the bond to be cleaved. Both of these classes of endoglycosidases are enzymes that cleave *β*-endo-N-acetylglucosamine bonds. The prokaryotic enzymes have specificity for HA, while the eukaryotic enzymes are also able to cleave chondroitin sulfate, albeit at a much slower rate, and (3) a curious class of hyaluronidases that are *β*-endoglucuronidases used by leeches and other invertebrates. Much less is known about this class of enzyme.

 Eukaryotic hyaluronidases were until recently relatively neglected enzymes, largely because of the difficult in purifying them. Hyaluronidases are extremely unstable in the absence of detergents and protease inhibitors. They occur in very low concentrations, with activities one log greater than other globular enzyme proteins. The concentration of hyaluronidase in human plasma is 60 ng/mL [34].

 There are six hyaluronidase-like sequences in the human genome, while rodents have seven such sequences. All are transcriptionally active with unique tissue distributions. In the human, three genes (*HYAL1, HYAL2*, and *HYAL3*) are found tightly clustered on chromosome 3p21.3. Another three genes (*HYAL4, PHYAL1 *(a pseudogene), and PH20, sperm adhesion molecule1 (*SPAM1*)) are clustered similarly on chromosome 7q31.3 [35].

 The enzymes HYAL1 and 2 constitute the major hyaluronidases in somatic tissues; HYAL1, an acid-active lysosomal enzyme, was the first somatic hyaluronidase to be isolated and characterized. Why an acid-active hyaluronidase should occur in plasma is not clear. HYAL1 is able to utilize HA of any size as a substrate and generates predominantly tetrasaccharides. HYAL2 is also acid active, anchored to plasma membranes by a GPI (glycosylphosphatidylinositol) link.

 The schema for HA catabolism [36, 37] indicates that HMW HA is taken up by cells by the concerted effort of the receptor CD44, the enzyme HYAL2, and the proton pump Na^+^H^+^ exchanger-1 (NHE1). An acidic microenvironment is created on the surface of the cell in membrane invaginations termed “lipid rafts” [38]. HYAL2 cleaves the HMW HA to a limit product of approximately 20 kDa, or about 50 disaccharide units. The resulting HA fragment is delivered to early endosomes and to lysosomes wherein the lysosomal enzyme HYAL1 cleaves the HA further to predominantly tetrasaccharide fragments. HYAL1, in the artificial test tube environment, can accept any sized HA polymer as a substrate and degrade it to the limit tetrasaccharide. The additional activities of the lysosomal exoglycosidases, *β*-glucuronidase, and *β*-hexosaminidase cleave fragments to single hexose sugars that then exit lysosomes to enter cytosolic pathways.

 Not all tissues that contain HYAL1 activity synthesize that enzyme. Active endocytosis of the protein from the circulation occurs [39]. Monocytes contain no mRNA for HYAL1, yet have very high levels of enzyme activity (unpublished observations). Megakaryocytes and platelets contain no HYAL1 [40], perhaps because they lack the receptors for endocytosis of circulating HYAL1.

 Evidence from an unexpected quarter sheds light on this anomalous situation. In the lysosomal storage disease termed X-disease, the mannitol-6-P receptors for lysosomal enzymes are defective. In such cultured fibroblasts, most lysosomal enzymes are absent, but levels of HYAL1 are normal. This suggests that the HYAL1 is taken by an unknown and, as yet unidentified receptor, one that is not affected by the lesion in the mannose-6-P receptor pathway. From this, it can be deduced that the unknown receptor is absent or blocked in platelets and megakaryocytes.

 These hyaluronidases are endoglycolytic activities with specificity for the *β*1-4 glycosidic bond. These enzymes have a hydrolytic mechanism of action, compared to the eliminase mechanism of prokaryotes. Isolation and characterization of the vertebrate enzymes indicate that they are ubiquitous. Rapid progress in genome analyses has provided much information. There are six hyaluronidases in the human genome [41, 42] with seven being present in the murine genome. Two hyaluronidases, HYAL1 and HYAL2, are the predominant activities in somatic tissues, working in concert to degrade HMW polymers to tetrasaccharides as the predominant end product.

 HYAL1 is an acid-active lysosomal enzyme and the first to be isolated and characterized [34, 43, 44]. HYAL1 is a 57 kDa polypeptide that also occurs in a processed 45 kDa form, the result of two endoprotease reactions, disulfide bonds binding the resulting two chains together. These two forms do not have a zymogen-active enzyme relationship, since they have similar specific activities. The fragment generated by the cleavage reactions may also have an important function in the overall economy of the niche.

 An inactive 70-kDa-precursor form of HYAL1 is present in some mammalian sera, including calf serum, as can be observed on electrophoretic gels by Western blot. The inactive unprocessed form occurs in sera from many species in which it is claimed that no acid-active enzyme exists [45].

 It is not clear why an acid-active enzyme is present in the circulation. Some animals have no apparent activity in serum or plasma. At one time, it was assumed that the serum enzyme, now known to be HYAL1, could not be an important activity if it was entirely absent in some mammalian species. However, it has been demonstrated that an inactive high molecular form occurs, as shown by Western blot in gels of such sera (R. Stern, unpublished observations).

 HYAL2 has several forms, one being anchored to the external surface of plasma membranes by a GPI-link. HYAL2 also occurs in a processed soluble form, and a portion can be detected in early lysosomes. The several forms are thought to shuttle between various cellular compartments in the process of HA internalization and degradation, as it is delivered from the extracellular space in steps to the final destination in acid lysosomes. HYAL2 cleaves the large HA polymer to a limit product of 20 kDa, or about 50 disaccharide units, while HYAL-1 *in vitro* is able to digest any HA chain including the high-molecular-weight polymer to the tetrasaccharide fragment.

 While only two hyaluronidases participate in HA catabolism in normal somatic tissues, aberrant expression of other hyaluronidases can be detected in cancerous tissues. One of the products of the six hyaluronidase-like sequences in the human genome, PH-20, a neutral-active hyaluronidase, was originally assumed to be sperm specific. Using more sensitive techniques, the enzyme can be detected in other tissues, in the epididymis, seminal vesicles, and prostate gland [46]. Additionally, PH20 can be aberrantly expressed in a number of human malignancies [47–50].

### 4.2. Nonenzymatic Cleavage

A portion of the breakdown of HA takes place by oxidation reactions. The cleavage is caused by unstable molecules, by reactive oxygen species (ROS) and by free radicals [51, 52]. The proportions of HA catabolism between enzymatic and oxidative cleavage reactions have not been established, though it is assumed that the former predominates. These proportions may vary considerably, depending on the physiological situation. Early in acute inflammation, the myeloperoxidase enzyme reaction associated with neutrophils may be an important catalytic mechanism for HA breakdown.

 There are major differences between the products of enzymatic and free radical catabolism. While enzymatic cleavage results in polymers that are identical in structure to the parent polymer, oxidative cleave generates polymers with oxidized termini. Such structural differences may provide metabolic cues. It is possible to determine the proportion of HA breakdown enzymatic and nonenzymatic mechanisms, based on such differences between their respective reaction products. However, to date, there has been no research performed to address this issue, despite its intrinsic importance in biology. ROS are constantly being produced in the body and are tightly regulated to maintain a redox balance or homeostasis, together with a series of antioxidants and attendant reactions, for example, glutathione, superoxide dismutase and the hexose monophosphate shunt. HA, hyaluronidases, and these redox mechanisms constitute interactions between a number of physiological situations as well as the pathophysiology of infection, inflammation, and the immune responses. This is an important area of biology also that has been relatively neglected, although inroads are currently being undertaken [53].

## 5. Hyaluronan Receptors

There are an ever-growing number of HA receptors. A conundrum to be dealt with is the indistinct boundary between membrane-bound receptors and HA-binding proteins, known as hyaladherens. The prominent receptor, CD44, also occurs as a soluble circulating protein, perhaps functioning as a decoy for the cell-bound receptor.

### 5.1. CD44

The CD (clusters of differentiation) system is commonly used as cell markers in immunophenotyping. CD44 is considered the most prominent receptor for HA. It is a transmembrane glycoprotein that occurs in a wide variety of isoforms, products of a single gene with expression of 10 variant exons [54]. The CD44 isoform can occur with the total absence of variant exon insertions, referred to as CD44S or the standard form. The variant exons are all inserted in various combinations into a single extracellular position near the membrane insertion site. Additional variations in CD44 structure occur as a result of posttranslational glycosylation, addition of various GAGs, including chondroitin sulfate and heparan sulfate. CD44 is widely distributed, being found on virtually all cells except red blood cells.

 CD44 plays a major role in myriads of physiological processes [55, 56] and is particularly important in malignancy, in tumor cell homing and metastasis. There is a vast literature on the combinations of variant exons that occur on tissue-specific cancers.

### 5.2. RHAMM

RHAMM is also given the designation CD168. This protein exists in the nucleus and cytoplasm, as well as on the cell surface. This receptor binds to the mitotic spindle, participates in cell locomotion, focal adhesion, and contact inhibition. It also has many functions that support the transformed state [57], levels being over-expressed in many human cancers. To designate it as a receptor only is obviously a simplification. Many of the proteins associated with HA metabolism have multiple functions. The names scientists apply to these proteins can be misleading, as the assumption is often made that their names designate their only function.

 RHAMM can partner with CD44 on cell surfaces where it enhances CD44-mediated signaling. It thus contributes to tumor progression by enhancing tumor-promoting properties of CD44. The close coordination of the RHAMM and CD44 receptors is further supported by the observation that mice with a genetic deletion of CD44 upregulate expression of RHAMM, the latter being able to substitute for many of the functions usually carried out by CD44 [58].

### 5.3. HARE

HARE (HA receptor for endocytosis), also known as stabilin-2, binds and internalizes not only HA and chondroitin, but also sulfated GAGs such as chondroitin sulfate, dermatan sulfate, and heparin [59].

### 5.4. LYVE-1

LYVE-1 stands for (lymphatic vessel endothelial HA receptor)-1. This cell surface receptor on lymphatic endothelial cells for HA is the subject of debate, but its conservation in evolution indicates that it is an important molecule [60]. LYVE-1 is not restricted to lymphatic vessels, but is also expressed in liver sinusoids, suggesting that it is involved in HA drainage in lymphatics and liver, and therefore in overall body turnover.

 An unexpected complexity is the regulation of LYVE-1 HA-binding activity by glycosylation and sialylation. This receptor, like CD44, may become active only after appropriate unmasking *in vivo* [61].

### 5.5. TLR-2 and -4

TLR (toll-like receptors) -2, and -4, CDs 282 and 284 respectively, are membrane receptors responsible for initiating action from cells of the innate immune system by stimulation of pathogen-associated molecular patterns (PAMPs) [62, 63]. TLR-2 is present on macrophages where, when stimulated by gram-positive bacteria, it induces transcription factor NF-KappaB and thereby the release of proinflammatory cytokines. TLR-4 is also found on macrophages where the stimulation is induced by lipopolysaccharide on gram-negative bacteria. These in turn initiate signal transduction pathways within macrophages. Since the cellular coat of both *Streptococcus* A and C contain HA components, data suggests that short chain HA may function as a PAMP in the inflammatory environment. This signal transduction has been demonstrated in lung and epithelial tissue in both infectious and traumatic situations [60–64].

## 6. Chain Length as an Information-Rich System

Despite its simple repeating structure, HA has a wide range and occasionally contradictory functions, even though it is without branch points and without sulfation or other secondary modifications. The multiple functions can be attributed to differences in chain length. The variation in size is an extraordinarily rich informational system [65, 66]. In general, high-molecular-weight HA occurs in normal healthy tissues. Fragmented HA, on the other hand, is highly inflammatory, angiogenic, and immune stimulatory, a reflection of tissues under stress. The large HA polymers are, in marked contrast, anti-inflammatory, anti-angiogenic, and immunesuppressive.

 Hyaluronan is critical in edema, inflammation, and during wound healing. Polymer size is critical throughout these processes. Tissue edema is one of the five cardinal signs of inflammation. Such swelling is comprised predominantly of HA, a concept that is not sufficiently appreciated. Indeed, many of the inflammatory cytokines induce HA production, and, conversely, HA fragments induce such cytokine synthesis in a self-stimulatory cycle [55, 67]. Hyaluronan, in a size-specific manner, also modulates a great number of immune responses [68].

## 7. The Malignant Connection

Hyaluronan plays a critical role in malignancy [69–71], levels of HA often correlating with tumor aggressiveness and poor prognosis. Such correlations have been validated for both epithelial tumors (carcinomas), as well as for malignancies of mesenchymal origin (sarcomas). Of particular interest is the observation that the HA content of the nonmalignant stromal component of tumors also correlates with malignant aggressiveness, suggesting that the stromal component is not entirely normal. Tumors may commandeer stromal populations from bone marrow or induce expansion of stem cell-like or populations of fibroblasts that resemble embryonic fibroblasts.

 The balance of synthesis and degradation of HA is regulatory for cancer cell proliferation and motility *in vitro*, and in tumor invasion and progression *in vivo* [72–76]. Of particular importance is the essential role of HA in the microenvironment during the process of tumor initiation and early progression [76]. As mentioned, the importance of local stroma in supporting such events, and peculiarities of the attendant HA metabolism is critical for success of the cancer. A recent volume profiles the many functions of HA in cancer biology and in the transformed state [77].

## 8. Hyaluronan in Skin

More than 50% of total body HA is contained in skin and thus requires “special handling.” It is responsible for skin hydration, and protects skin against free radical damage, particularly against UV light, both UVA and UVB.

 There are many reports that document decreasing levels of skin HA with aging, with the suggestion that this is responsible for the deteriorating appearance of skin with aging. Such observations are based on histochemical staining criteria. However, biochemical extraction indicates that HA levels remain constant with age. The HA becomes increasingly tissue associated, becoming progressively more resistant to extraction as a function of age [78].

 The apparent results of the histochemical investigations can be explained by increasing competition between tissue proteins for HA binding sites and the HA-binding peptide as a function of age. The HA, encased within tissue proteins may be restricted from functioning as a hydrating molecule. This proviso also indicates that the HA-staining procedure, as normally performed with an HA-binding peptide, is not a quantitative procedure.

 Hyaluronan in skin occurs in both dermis and epidermis, with dermis containing the greater proportion. Epidermal HA is more loosely associated and more easily extracted from tissue. Formalin, an aqueous fixative, easily removes most HA from epidermis and is less able to extract HA from dermis. Alcoholic acid formalin enhances histolocalization of epidermal HA, and indicates that considerable levels are contained therein [79].

 Skin HA has a very rapid turnover, with a half-life of 1-2 days in the epidermis [80]. The turnover rate in the dermis is similar, with catabolism occurring in liver and lymph nodes, following lymphatic drainage [31]. The turnover mechanism in the epidermis is not clear and may be a combination of free radical fragmentation stimulated by UV light, and enzymatic degradation.

 Hyaluronan is most prominent in the upper spinous and granular, where much of it is extracellular. In the basal layer, HA is predominantly intracellular and is not easily eluted out during aqueous fixation. Basal keratinocyte HA is involved in mitotic events, presumably, while extracellular HA in the upper layers of the epidermis is involved in barrier disassociation and sloughing of cells.

 Hyaluronan of the epidermal ECM forms two different structures; a pericellular coat close to the plasma membrane, forming the pericellular matrix, and HA chains that coalesce into large cables. Such cables, with induced expression under inflammatory events, bind leukocytes, whereas the pericellular HA does not [28, 81].

 The HA content of the dermis is far greater than that of the epidermis. The papillary dermis has more prominent levels of HA than the reticular dermis. Exogenous HA is cleared from the dermis and rapidly degraded [31]. The dermal fibroblast provides the synthetic machinery for dermal HA. What makes the papillary dermal fibroblast different from other fibroblasts is not known. However, these cells have an HA synthetic capacity similar to that of the fibroblasts that line joint synovium, responsible for the HA-rich synovial fluid, or the hyalocytes of the eye that provide the vitreous of the ocular chambers.

## 9. Recent Additions to the Many Functions of HA

Despite the fact that HA does not give up its secrets easily, new functions for HA continue to emerge. An interesting connection has been described between HA and autophagy. Hyperglycemia is one of many factors that induces autophagy. Cells cultured in hyperglycemic medium initiate a stress response that includes HA synthesis. The HA is extruded into the ECM in a structural form recognized by inflammatory cells [29]. Cyclin D3 is central to such events. Abnormal deposits of HA and cyclin D3 occur in diabetic rat glomeruli, indicating that a similar process occurs *in vivo* [82]. More importantly, the HA stress response and autophagy may be major contributors to the multiple pathologies associated with diabetes [28].

 Matrix HA also has a dramatic effect in immune regulation, on the phenotype of regulatory T cells [83]. The HA-containing ECM functions as a biosensor for the inflammatory microenvironment important for immune tolerance. HA that is HMW promotes induction of IL-10-producing regulatory T cells from T-cell precursors, while fragmented HA does not. This has been demonstrated in an IL-10-dependent mouse colitis system, in which HMW HA suppresses disease through such T-cell induction. The ability of HMW HA to cross-link CD44 on the cell surface may be an underlying mechanism for this phenomenon [84], thereby maintaining immunological tolerance.

## 10. Conclusion

There remain many questions regarding this highly ionic as well as highly ironic polymer. How do cell response mechanisms distinguish between the different sizes of linear HA polymers, given the identity of their chemical structures? One intuitive answer is that various HA chain sizes take on different physical-chemical configurations and that the sensing mechanisms respond to such differences. Another possibility is that different-sized HA fragments can associate with different profiles of hyaladherens or that there are major changes in affinity, depending on HA size. It is probably a combination of all such factors that determine the differences in responses.

## Abbreviation

CD: Clusters of differentiation
ECM: Extracellular matrix
GAG: Glycosaminoglycan
GPI-linked: Glycosylphosphatidylinositol-linked
HA: Hyaluronan, hyaluronic acid
HARE: HA receptor for endocytosis
HAS: HA synthase
*HAS*: The gene that codes for a HAS
HMW: High molecular weight
HYAL: Hyaluronidase
*HYAL*: The gene that codes for a hyaluronidase
LYVE-1: Lymphatic vessel endothelial HA receptor
NHE1: Na^+^H^+^ exchanger-1
NF-KappaB: Nuclear factor kappa-light-chain-enhancer of activated B cells
PAMP: Pathogen-associated molecular pattern
*PHYAL*: A pseudogene for a hyaluronidase
RHAMM: Receptor for HA-mediated motility
ROS: Reactive oxygen species
TLR: Toll-like receptor
TSG-6: Tumor-necrosis-factor-stimulated gene 6
UV: Ultraviolet.
